# Neutralizing antibody levels detected early after mRNA-based vaccination do not predict by themselves subsequent breakthrough infections of SARS-CoV-2

**DOI:** 10.3389/fimmu.2024.1341313

**Published:** 2024-02-09

**Authors:** Roberto Alonso, Sergio Gil-Manso, Pilar Catalán, Ignacio Sánchez-Arcilla, Marco Marzola, Rafael Correa-Rocha, Patricia Muñoz, Marjorie Pion, the Gregorio Marañón Microbiology-ID COVID-19 Study Group

**Affiliations:** ^1^ Department of Clinical Microbiology and Infectious Diseases, Hospital General Universitario Gregorio Marañón, Madrid, Spain; ^2^ Instituto de Investigación Sanitaria Gregorio Marañón, Hospital General Universitario Gregorio Marañón, Madrid, Spain; ^3^ CIBER (Centro de Investigación Biomédicas en Red) de Enfermedades Respiratorias, CIBERES, Barcelona, Spain; ^4^ Department of Medicine, Facultad de Medicina, Universidad Complutense de Madrid, Madrid, Spain; ^5^ Advanced ImmunoRegulation Group, Instituto de Investigación Sanitaria Gregorio Marañón, Hospital General Universitario Gregorio Marañón, Madrid, Spain; ^6^ Department of Labour Risks Prevention, Hospital General Universitario Gregorio Marañón, Madrid, Spain; ^7^ Laboratory of Immune-Regulation, Instituto de Investigación Sanitaria Gregorio Marañón, Hospital General Universitario Gregorio Marañón, Madrid, Spain

**Keywords:** SARS-CoV-2, anti-RBD IgG, neutralizing antibodies, breakthrough infection, humoral immunity

## Abstract

The development of mRNA vaccines represented a significant achievement in response to the global health crisis during the SARS-CoV-2 pandemic. Evaluating vaccine efficacy entails identifying different anti-SARS-CoV-2 antibodies, such as total antibodies against the Receptor Binding Domain (RBD) of the S-protein, or neutralizing antibodies (NAbs). This study utilized an innovative PETIA-based kit to measure NAb, and the investigation aimed to assess whether levels of anti-RBD IgG and NAb uniformly measured 30 days after vaccination could predict individuals at a higher risk of subsequent infection in the months following vaccination. Among a cohort of healthy vaccinated healthcare workers larger than 6,000, 12 mRNA-1273- and 115 BNT162b2-vaccinated individuals contracted infections after the first two doses. The main finding is that neither anti-RBD IgG nor NAb levels measured at day 30 post-vaccination can be used as predictors of breakthrough infections (BI). Therefore, the levels of anti-SARS-CoV-2 antibodies detected shortly after vaccination are not the pivotal factors involved in antiviral protection, and other characteristics must be considered in understanding protection against infection. Furthermore, the levels of anti-RBD and NAbs followed a very similar pattern, with a correlation coefficient of r = 0.96. This robust correlation would justify ceasing the quantification of NAbs, as the information provided by both determinations is highly similar. This optimization would help allocate resources more efficiently and speed up the determination of individuals’ humoral immunity status.

## 1 Introduction

The mRNA-based vaccines against COVID-19 have been a key tool to control the pandemic, reducing the number of infections and symptomatology in those vaccinated and later infected. mRNA vaccines confer robust humoral and cellular immunity (1, 2), and thanks to their design, its mRNA sequence may be modifiable to adapt to the mutations present in the new variants of concern (VOCs) (3). During the early stages of the pandemic, some individuals with newly SARS-CoV-2 infections were reported after the administration of the first two doses of the mRNA vaccines, referred to as Breakthrough Infections (BI) (4). These posed a significant public health risk in pandemic control, and therefore, identifying factors that could predict which vaccinated individuals might be at a higher risk of infection after vaccination could allow for the implementation of control and containment measures to reduce the number of BI.

To assess the antiviral immune status, the detection of specific antibodies against S-protein of the SARS-CoV-2 conferred after vaccination has been widely used world-wide. The main techniques to determine such specific antibodies from vaccinated volunteers were ELISA or Chemiluminescence Immunoassay (CLIA)-based kits. Among all types of antibodies, neutralizing antibodies (NAbs) are a special type with the ability to block specifically the interaction between the receptor-binding domain (RBD) of the SARS-CoV-2 and its primary receptor in humans, the Angiotensin-Converting Enzyme 2 (ACE-2), distributed in multiple cell types and tissues throughout the body (5). However, determining the neutralization capacity of antibodies typically requires facilities with a high level of biosecurity, co-cultures with live or attenuated virus, and is often unfeasible for a large number of samples. Recently, a new kit for the determination of NAbs has been developed, and already used in the work of Fogolari et al. (6). This kit is based on the Particle Enhanced Turbidimetric Immunoassay (PETIA) technology and relies on a two-steps procedure. The first step consists of the incorporation of a latex-coated recombinant RBD antigen of the SARS-CoV-2, which forms complexes with anti-RBD antibodies conferred after natural infection or vaccination. Subsequently, latex particles coated with ACE-2 are added. The kit is designed to determine the competition between ACE-2-coated particles and neutralizing antibodies anti-RBD to bind the RBD antigen. Therefore, the kit quantitatively determines the number of antibodies with neutralizing capacity.

Therefore, using this innovative kit, we aimed to study whether the levels of anti-SARS-CoV-2 antibodies (anti-RBD IgG or NAb) conferred by the first two doses of the mRNA vaccines uniformly measured 30 days after vaccination, could be a predictor factor of those individuals protected from or at risk of breakthrough infection.

## 2 Methods

This study was approved by the research ethics committee of the Hospital General Universitario Gregorio Marañón (HGUGM) (MICRO.HGUGM.2020-021) and was performed according to the principles of the Declaration of Helsinki and the European Union Regulation 2016/679. All samples in this study were obtained in March 2021, and all the individuals who participated in the study were followed up until December 2021, just before the administration of the third dose of the mRNA vaccines and the apparition of the SARS-CoV-2 Omicron variant (B.1.1.529).

Healthcare workers of the HGUGM were vaccinated with the first two doses of the mRNA-based vaccines between January and February 2021: BNT162b2 (Pfizer/BioNTech) or mRNA-1273 (Moderna). After the administration of both doses, a seroprevalence study was performed 30- and 240-days post-vaccination to assess seropositivity and anti-RBD IgG levels post-vaccination (7). A total of 6,124 vaccinated healthcare workers participated in the seroprevalence study at day 30 post-vaccination, 954 of them vaccinated with mRNA-1273 and 5,170 vaccinated with BNT162b2 (corresponding to 15.58% and 84.42% of the total participants, respectively). Among these volunteers, between March and December 2021 (prior to the third dose and the appearance of the Omicron variant), breakthrough infections (BI) were reported, defined as infections by SARS-CoV-2 in subjects with no previous history of COVID-19, vaccinated with the first two doses of mRNA-based anti-SARS-CoV-2 vaccines. A total of 12 BI cases were reported in the mRNA-1273-vaccinated and 115 in BNT162b2-vaccinated healthcare workers’ cohorts (1.28 and 2.22% of total vaccinated per vaccine type, respectively). To compare the humoral status of BI cases with non-infected volunteers during the follow-up period (control, CT), we randomly selected 22 and 25 healthy volunteers vaccinated with mRNA-1273 or BNT162b2, respectively.

All blood samples were uniformly obtained at 30 days post-vaccination, and individuals were followed up until December 2021, classifying individuals in the CT or BI cohorts. Serum samples were obtained by centrifugation of blood, and cryopreserved until the quantification of antibodies. Both antibody determinations were performed using an ARCHITECT i2000 instrument (Abbott; Chicago, USA). Anti-RBD IgG antibodies were determined using the quantitative CLIA SARS-CoV-2 IgG II Quant Reagent Kit (Abbott), and the NAb antibodies were determined using the PETIA SARS-CoV-2 Neutralizing Antibodies Kit (SGM Italy; Rome, Italy). NAb antibodies were expressed as AU/ml, and anti-RBD IgG antibody levels were standardized into BAU/ml (1 BAU/ml = 0.142 x AU/ml), using the previously established conversion criteria (8–10).

The statistical analysis and the figures were created using SPSS version 25 (IBM; NY, USA) and GraphPad Prism version 9.0 (GraphPad Software, Inc; California, USA). The respective statistical tests are indicated in the legend of each of the figures. Statistical differences were considered when p value < 0.05.

## 3 Results

### 3.1 Individual’s characteristics

Between the seroprevalence study performed in March 2021, 30 days after first two doses of mRNA-based vaccines, and the apparition of the Omicron variant in December 2021, a total of 127 BI cases were confirmed at the Department of Labor Risks Prevention (DLRP) of the HGUGM, 12 of them previously vaccinated with mRNA-1273 and 115 with BNT162b2. Taking into account that the seroprevalence study involved 5,170 volunteers vaccinated with BNT162b2 and 954 vaccinated with mRNA-1273, BI cases accounted for 2.22% of BNT162b2-vaccinated and 1.28% of mRNA-1273-vaccinated individuals participating in the study. We randomly selected 22 volunteers vaccinated with mRNA-1273 and 25 vaccinated with BNT162b2 vaccines, who participated at the seroprevalence study of the HGUGM and did not report any positive test for SARS-CoV-2 detection, nor COVID-19 related symptomatology (**Table 1**). The study was performed during the dominant period of the Alpha- (B.1.1.7) and Delta- (B.1.617.2) variants, and these variants were determined in some BI cases. Comparing all individuals, no significant differences were found for age and gender distribution. In the case of the BI groups, there were no significant differences for the SARS-CoV-2 variants causing the infection or for the days elapsed between humoral immunity determination and infection. To facilitate the understanding of the study design, an outline of the stages carried out and the corresponding months is shown in **Supplementary Figure 1**: 1) volunteers were vaccinated in January and February 2021, 2) at day 30 post-vaccination (March 2021), serum samples were uniformly obtained for the determination of anti-RBD IgG and NAb levels in all subjects, and 3) between March and December 2021, subjects were followed up, identifying BI cases at DLRP. For each subject in the BI groups, the time elapsed between antibodies quantification and BI was calculated (days between steps 2 and 3).

**Table 1:** Individual’s characteristics.

	mRNA-1273 BI	mRNA-1273 CT	BNT162b2 BI	BNT162b2 CT	*p-value*
Number individuals	12	22	115	25	
Gender, (%)					0.928
Male	3 (25)	4 (18)	23 (20)	6 (24)	
Female	9 (75)	18 (82)	92 (80)	19 (76)	
Age, mean (± SEM)	44.75 (± 3.10)	49,45 (± 2.10)	41.94 (± 1.19)	44.48 (± 2.46)	0.056
Type of variant					1.000
Unknown variant	6	–	56	–	
Delta (B.1.617.2)	5	–	48	–	
Alpha (B.1.1.7)	1	–	11	–	
Days between antibodies detection and infection, mean (± SEM)	145.90 (± 12.17)	–	165.60 (± 6.34)	–	0.233

Individuals are divided into 4 cohorts attending to their vaccine type and infection status: mRNA-1273 BI, mRNA-1273 CT, BNT162b2 BI and BNT162b2 CT. For each group, it is detailed the number of individuals, the gender, the mean age, and in the BI groups, when it was determined, the variant responsible of the infection. Also, it is indicated in days ± Standard Error of the Mean (SEM), the time elapsed between antibodies detection and the infection. To analyze gender and type of variant, chi-squared test was used. Mann-Whitney test was used to compare time elapsed between antibodies detection and infection between the BI groups, and Kruskal Wallis test was used to compare volunteers age at the time of the study. Statistical differences were considered when p value < 0.05.

### 3.2 Antibodies detected early after vaccination cannot predict individuals susceptible of breakthrough infections

**Figure 1:**
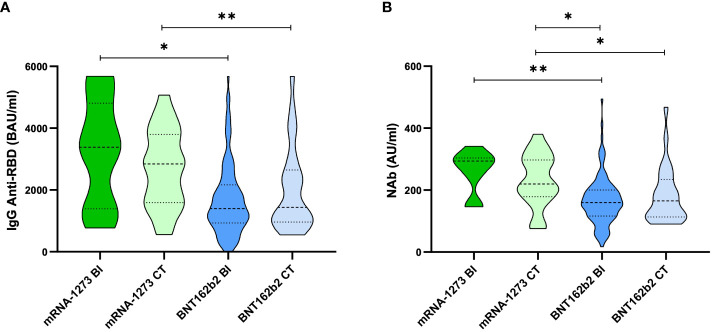
Anti-RBD IgG and NAb quantified in serum samples 30 days post-vaccination. Violin plots representing **(A)** anti-RBD IgG BAU/ml and **(B)** NAb AU/ml per group, split per vaccine type and infection status: mRNA-1273-CT (light green), mRNA-1273-BI (dark green), BNT162b2-CT (light blue) and BNT162b2-BI (dark blue). The thick dotted line within the violin plot indicates the median and the thin dotted line indicates the quartiles (25 and 75%). For the anti-RBD IgG, the dotted line at 5,680 BAU/ml indicates the upper limit of quantification (ULOQ). For statistical analysis, Kruskal-Wallis test was performed. *p value < 0.05. **p value < 0.01.

Anti-RBD IgG levels were measured in all the subjects included in the study (n = 174; 12 BI mRNA-1273, 115 BI BNT162b2, 22 CT mRNA-1273, and 25 CT BNT162b2 ). NAb levels were measured in all individuals except for 3 individuals of the BI mRNA-1273 group and 3 individuals of the BI BNT162b2 group in which the anti-RBD IgG levels were measured but NAb levels could not be assessed due to an insufficient amount of sample. As already described in multiple studies, median levels of anti-RBD IgG were higher in individuals vaccinated with mRNA-1273 (3380 ± 501 BAU/ml ± SEM in the BI group, and 2842 ± 269 in the CT group) compared to those vaccinated with BNT162b2 (1398 ± 107 BAU/ml ± SEM in the BI group, and 1436 ± 275 in the CT group). No significant differences were observed comparing BI and CT groups (**Figure 1A**). Since total anti-RBD IgG encompasses a large number of antibody types, we compared the levels of antibodies with neutralizing capacity (NAb) in the CT and BI groups for each vaccine type. The distribution of NAb levels was very similar to that of anti-RBD IgG; mRNA-1273-vaccinated individuals presented higher median levels (294 ± 23 and 219 ± 18, AU/ml ± SEM in BI and CT groups, respectively) compared to BNT162b2 (160 ± 7 and 165 ± 19, AU/ml ± SEM in BI and CT groups, respectively) (**Figure 1B**). As observed for anti-RBD IgG, no statistical differences were observed between CT and BI groups within the same vaccine type. In fact, the mRNA-1273 BI cohort presented slightly higher levels of anti-RBD IgG, and especially NAb, compared to the CT cohort. Based on these findings, it can be concluded that assessing anti-RBD and NAb levels 30 days post-vaccination is not a reliable factor for predicting which individuals are at higher risk of subsequent infection after vaccination.

### 3.3 Robust correlation between anti-RBD IgG and NAb levels

**Figure 2:**
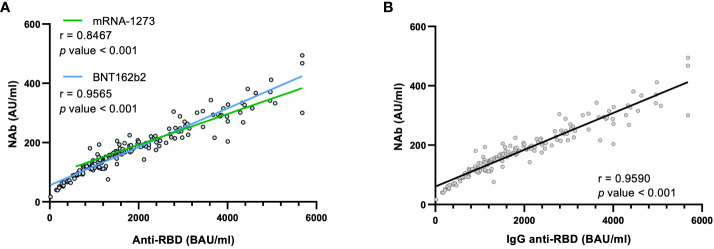
Correlations between anti-RBD IgG and NAb levels. **(A)** Correlation between both types of antibodies attending to the two types of vaccines or **(B)** independently of the vaccine type. Each point corresponds to a single determination, and the bold line indicates the linear regression. The r is the Spearman Correlation Coefficient (SCC). Statistical differences were considered when p value < 0.05.

As described in **Figure 1**, the distribution of anti-RBD IgG and NAb levels in each of the 4 cohorts was very similar. Therefore, we correlated both determinations, and observed that anti-RBD IgG and NAb levels were strongly correlated for both vaccines: mRNA-1273 (r = 0.8467, p value < 0.001) and BNT162b2 (r = 0.9565, p value < 0.001) (**Figure 2A**). Regardless of the type of mRNA-based vaccine, the Spearman Correlation Coefficient (SCC) was very robust too (r = 0.9590, p value < 0.001) (**Figure 2B**). Given the robust correlations obtained, this would allow us to conclude that the quantification of NAb may not be necessary since it would be an expensive, time-consuming and redundant measure compared to the anti-RBD IgG determination. Therefore, to assess humoral immunity after vaccination, quantifying only anti-RBD IgG would be enough to determine the humoral status.

### 3.4 Antibody levels also cannot predict when breakthrough infections will occur

**Figure 3:**
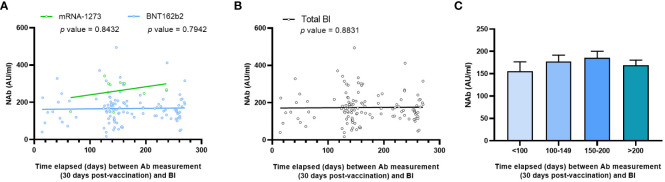
Correlations between NAb levels and time elapsed between NAb quantification and infection. **(A)** Correlation attending to the two types of vaccines or **(B)** independently of the vaccine type. Each point corresponds to a single determination, and the bold line indicates the linear regression. NAb is indicated in AU/ml, and time elapsed between NAb quantification and infection is indicated in days. **(C)** NAb levels divided into different cohorts taking into account time from vaccination to infection. Statistical differences were considered when p value < 0.05.

Although neither anti-RBD IgG nor NAb levels proved to be reliable predictors of BI cases, we hypothesize that NAb levels may impact the time elapsed between vaccination and SARS-CoV-2 infection in the BI group. Therefore, we hypothesized that low levels of NAbs detected uniformly 30 days after vaccination could be associated with worse protection and an early infection post-vaccination. We correlated both determinations taking into account the type of vaccine administered. We observed a SCC close to 0 for both vaccines; mRNA-1273: r = -0.0833, p value = 0.8432; and BNT162b2: r = 0.0364, p value = 0.7942 (**Figure 3A**). Regardless of the vaccine type, the overall correlation was very similar to those obtained previously in **Figure 3A**, with a SCC close to 0; r = 0.0191, p value = 0.8831 (**Figure 3B**). Due to the non-uniform timing of BI measurements, we stratified infected individuals based on the day of infection post-vaccination (**Figure 3C**). No differences in NAb levels were observed that could account for the duration of protection post-vaccination. Therefore, these results indicated that both NAb and anti-RBD IgG levels (data not shown) also failed to predict which BI individuals will be infected earlier or later.

## 4 Discussion

The COVID-19 pandemic has represented a shift in the global paradigm in recent years. Due to the uncertain worldwide situation, efforts have been made to develop vaccine strategies and understand the protection they provide against SARS-CoV-2 by studying multiple factors, being one of those the determination of specific antibodies against SARS-CoV-2, whether they are generated naturally after infection or through vaccination (11). While the determination of anti-RBD IgG is a cost-effective and a straightforward method that does not necessarily demand highly specialized personnel or complex techniques, the detected presence of these antibodies may not directly correlate with their antiviral functionality. Therefore, the specific determination of antibodies with neutralization capacity should provide a more comprehensive assessment of the protection, due to its direct implication in blocking the interaction between the S-protein of the SARS-CoV-2 and its receptor in humans, the ACE-2.

Fogolari et al. (6) had used the same PETIA-based kit for NAb and for anti-RBD IgG levels quantification, obtaining a correlation coefficient between them of r = 0.96, an identical value to the correlation that we obtained in this study. Therefore, we have independently demonstrated the robustness of both kits for determining anti-RBD IgG and NAb levels, and the strong correlation between both determinations. Unfortunately, our institution has biohazard restrictions in performing functional neutralization assays with wild-type SARS-CoV-2. Consequently, a direct comparison between the PETIA-based kit and established neutralization assays could not be conducted in our study. However, Fogolari’s study provides evidence of congruence between the results obtained from both assays. Moreover, this method is more cost-effective and time-saving compared to the standardized NAb determination process. Therefore, on the basis of all these results, we suggest that the assessment of anti-RBD IgG levels alone may adequately serve as a reliable indicator of humoral immunity status against COVID-19 in vaccinated individuals. This conclusion cannot be fully extrapolated to assess general anti-COVID-19 immunity, as the same correlation should be studied in terms of neutralizing capacity induced after natural infection.

BI have become pivotal in the context of the spread of SARS-CoV-2 and the generation of new variants. Multiple studies have assessed the vaccine effectiveness (VE) of anti-SARS-CoV-2 vaccines, describing a progressive and global decline in the VE, regardless of the vaccine used (12, 13). Also, several studies found that the neutralization capacity by using viral cultures is correlated with a protection from developing symptomatology in BI, but not assessing protection from infection (14–17). Like the decrease in anti-RBD IgG levels (7, 18), these studies have indicated a gradual decrease in NAb levels in the months following vaccination, and therefore, it can be assumed that this correlation between the two types of antibodies could be maintained over the months. This decline is correlated to an elevated risk of developing symptoms if infected, emphasizing the need for booster doses to restore NAb levels, as well as the need to continuously track immunity to assess the need for successive doses, as well as individuals at risk of becoming infected. Consequently, in our study, we first postulated that if low antibody levels are linked to a decline in VE and to an increase in symptomatology, they could also serve as a predictive indicator for vaccinated individuals with an inadequate vaccine response soon after vaccination, indicating a potential higher susceptibility to infection.

To evaluate this, we uniformly measured 30 days after vaccination both types of antibodies in a cohort of healthcare workers larger than 6,000 individuals, and we followed them during the first 8 months of vaccination, identifying several cases of BI. To our knowledge, this is the first work to evaluate the ability of anti-RBD IgG and NAb levels, measured by the novel PETIA-based kit, to be a predictor of the BI occurrence. We found higher levels of both types of antibodies (anti-RBD IgG and NAb) in the mRNA-1273-vaccinated individuals compared to the BNT162b2 cohort, a finding extensively reported and discussed in the literature previously (11, 18–20). However, we did not find differences between BI and CT groups, suggesting that neither of the two types of antibodies evaluated early after vaccination could serve as a predictor of future BI. As mentioned earlier, prior studies have indicated a decrease in total IgG levels over time (12, 13). Assessing these levels at the time of BI could provide valuable insights into the vaccine’s protection level. However, our study did not include this measurement as the spontaneity of SARS-CoV-2 infection after vaccination made it impossible to sample all individuals just prior to BI. Moreover, the guidelines of the Department of Labour Risks Prevention stipulated that BI individuals should stay at home without subsequent monitoring of their immunoglobulin levels.

The lack of predictive capacity of antibodies could suggest that in protecting and combating a SARS-CoV-2 infection post-vaccination not only the humoral immunity is involved, such as antiviral cellular immunity (21, 22), viral load, individual factors, vaccine regimen (23–26), and other yet-to-be-identified factors. During the pandemic, multiple test were developed to evaluate specific cellular response after stimulation with specific SARS-CoV-2 peptides of the fresh whole blood (27, 28) or previously purified PBMCs (29, 30). These studies widely described a wane in the cellular response after vaccination, in a manner similar to what occurs with humoral immunity, although with a smaller magnitude of decrease. In our research group, we have previously evaluated the cellular response and its correlation with the humoral response in a smaller cohort of vaccinated volunteers (11). We found that the BNT162b2 vaccine induced a modest correlation between humoral and cellular responses, whereas such correlation was not observed with the mRNA-1273 vaccine. Therefore, it can be assumed that the induction and coordination of the entire immune response may not be identical between the two vaccines, emphasizing the significance of investigating the entire immune system and its intrinsic interactions to comprehend the potential protection against this virus.

It is important to mention that in this work we have focused on a specific epidemiological period (during the predominance of the Alpha and Delta variants), studying only the effect of the first two doses of the original mRNA-based vaccines. One of the key questions for the future of controlling the pandemic is the ability to understand if mRNA vaccines adapted to new variants or other types of vaccines could induce a humoral and cellular response to control the new variants. In their work, Fogolari et al. also described that the correlation between anti-RBD IgG and neutralization capacity was maintained for Beta and Omicron variants (6). Therefore, despite the mutations across the sequence of the new emerging variants, this would suggest that the same results obtained in this manuscript could be obtained in the different epidemiological contexts generated by the new variants and new types of vaccines, although further monitoring should be done to confirm this hypothesis.

Finally, the methodology of the study presents some limitations that reduce the impact of the message conveyed. One of the limitations of this work is the lack of occupational information for the BI individuals, hindering our assessment of their exposure to SARS-CoV-2. Another limitation is the reduced number of BI cases, especially in the mRNA-1273-vaccinated cohort, which restricted our ability to draw more robust conclusions. Also, the follow-up period of 8 months limited partly the conclusions obtained in the study, and a longer-term follow-up should be necessary to deeply understand the anti-COVID-19 immunity after vaccinations and natural infections.

To conclude, this work holds significant clinical importance as it identifies two key points. First, the levels of total anti-RBD IgG and NAb do not serve as predictive markers for potential SARS-CoV-2 infections either early or late after vaccination (until 8 months). Hence, the concept of humoral protection must be used with caution. Second, the strong correlation between anti-RBD IgG and NAb levels suggests that determining anti-RBD IgG levels alone could sufficiently evaluate the generation of the humoral branch of the immune system post-vaccination. This finding is especially valuable for clinical routine, considering that determining the anti-RBD IgG level is simpler, quicker, and more cost-effective than measuring NAbs.

## Data availability statement

The raw data supporting the conclusions of this article will be made available by the authors, without undue reservation.

## Ethics statement

The studies involving humans were approved by Research Ethics Committee of the Hospital General Universitario Gregorio Marañón. The studies were conducted in accordance with the local legislation and institutional requirements. The participants provided their written informed consent to participate in this study.

## Author contributions

RA: Conceptualization, Formal analysis, Funding acquisition, Methodology, Project administration, Resources, Supervision, Validation, Visualization, Writing – original draft, Writing – review & editing. SGM: Formal analysis, Methodology, Investigation, Data curation, Validation, Visualization, Writing – original draft, Writing – review & editing. PC: Conceptualization, Visualization, Writing – review & editing. IS: Resources, Writing – review & editing. MM: Resources, Writing – review & editing. RC: Conceptualization, Funding acquisition, Investigation, Visualization, Writing – review & editing. PM: Conceptualization, Formal analysis, Funding acquisition, Project administration, Supervision, Validation, Visualization, Writing – review & editing, Resources. MP: Conceptualization, Data curation, Formal analysis, Funding acquisition, Investigation, Validation, Visualization, Writing – original draft, Writing – review & editing.

## Gregorio Marañón Microbiology-ID COVID-19 study group members

Luis Alcalá́, Teresa Aldámiz, Roberto Alonso, Beatriz Álvarez, Ana Álvarez-Uría, Alexi Arias, Elena Bermúdez, Emilio Bouza, Sergio Buenestado-Serrano, Almudena Burillo, Raquel Carrillo, Pilar Catalán, Emilia Cercenado, Alejandro Cobos, Cristina Díez, Pilar Escribano, Agustín Estévez, Chiara Fanciulli, Alicia Galar, M^a^ Dolores García, Darío García de Viedma, Paloma Gijón, Adolfo González, Helmuth Guillén, Jesús Guinea, Marta Herranz, Álvaro Irigoyen, Laura Vanessa Haces, Martha Kestler, Juan Carlos López, Carmen Narcisa Losada, Marina Machado, Mercedes Marín, Pablo Martín-Rabadán, Andrea Molero-Salinas, Pedro Montilla, Patricia Muñoz, María Olmedo, Belén Padilla, Rosalía Palomino-Cabrera, María Palomo, María Jesús Pérez-Granda, Daniel Peñas-Utrilla, Laura Pérez-Lago, Leire Pérez, Elena Reigadas, Cristina Rincón, Belén Rodríguez, Sara Rodríguez, Cristina ´Rodríguez-Grande, Adriana Rojas, María Jesús Ruiz-Serrano, Carlos Sánchez, Mar Sánchez, Amadeo Sanz-Pérez, Julia Serrano, Francisco Tejerina, Maricela Valerio, M^a^ CristinaVeintimilla, Lara Vesperinas, Teresa Vicente, Sofıa de la Villa.

## Supplementary material

Supplementary Figure 1 Schematic overview of the volunteer’s follow-up. The scheme outlines the initial vaccination schedule for mRNA vaccines, based in two consecutive doses administered with a 28-day interval for the mRNA-1273 vaccine (green) and a 21-day interval for the BNT162b2 (blue). Thirty days after the administration of the second dose, serum samples were collected from blood samples to quantify anti-RBD IgG and NAb. Volunteers were monitored until December 2021 and categorized into non-infected (control, CT) and breakthrough (BI) groups.
