# Supplementary figures and images for: Neutralizing antibody levels detected early after mRNA-based vaccination do not predict by themselves subsequent breakthrough infections of SARS-CoV-2
Source: Front Immunol. 2024 Feb 9;15:1341313. doi: 10.3389/fimmu.2024.1341313 (PMC10884961; doi:10.3389/fimmu.2024.1341313)

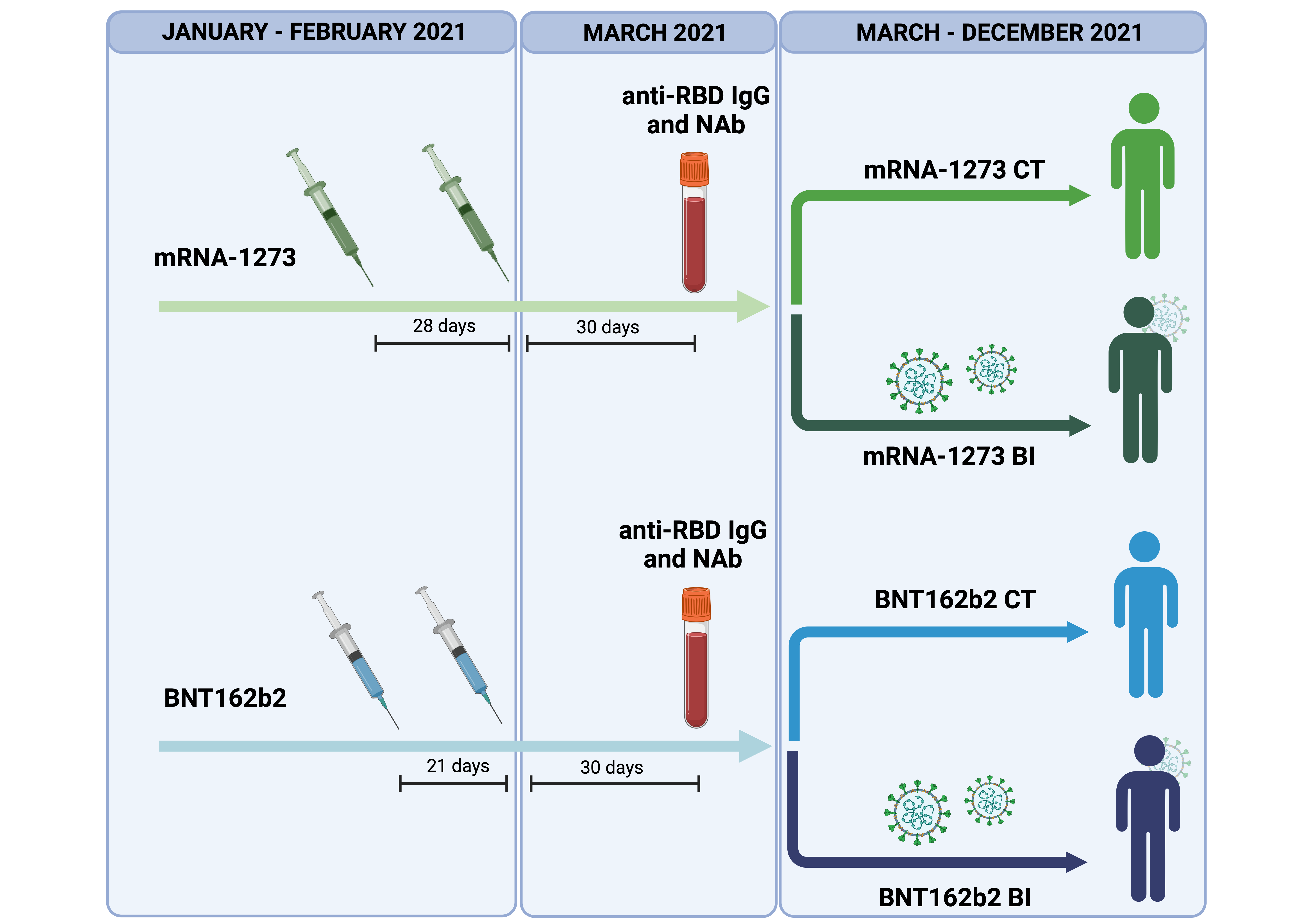

Supplement: Supplementary Figure 1 — Schematic overview of the volunteer’s follow-up. The scheme outlines the initial vaccination schedule for mRNA vaccines, based in two consecutive doses administered with a 28-day interval for the mRNA-1273 vaccine (green) and a 21-day interval for the BNT162b2 (blue). Thirty days after the administration of the second dose, serum samples were collected from blood samples to quantify anti-RBD IgG and NAb. Volunteers were monitored until December 2021 and categorized into non-infected (control, CT) and breakthrough (BI) groups. [file Image_1.png]
